# The causal relationship between obesity, obstructive sleep apnea and atrial fibrillation: a study based on mediated Mendelian randomization

**DOI:** 10.3389/fcvm.2024.1406192

**Published:** 2024-04-19

**Authors:** Tianyu Li, Li Rong, Yanlin Gao, Wei Cheng

**Affiliations:** Department of Cardiology, Anhui No.2 Provincial People's Hospital, Hefei, Anhui, China

**Keywords:** obesity, obstructive sleep apnea, AF, mediated Mendelian randomization, causal relationship

## Abstract

**Background:**

Atrial fibrillation (AF) is a common cardiac arrhythmia that is associated with obesity and obstructive sleep apnea syndrome (OSA). Obesity and OSA may increase the risk of AF by affecting cardiovascular health.

**Methods:**

The study used the Mendelian randomization (MR) approach, combined with two-sample and multivariable analyses, to assess the relationships between obesity, OSA, and AF. The study utilized GWAS data and applied various statistical methods for the analysis.

**Results:**

The study found that obesity increased the risk of OSA, which in turn significantly increased the risk of AF. Through mediating MR analysis, it was found that OSA played a certain role in the causal relationship between obesity and AF, with about 6.4% of the risk of AF being mediated by OSA.

**Conclusion:**

This study highlights the relationships among obesity, OSA, and AF, providing useful guidance for future clinical researches.

## 1 Introduction

Atrial fibrillation (AF) is a common cardiac arrhythmia characterized by rapid and irregular heartbeats, frequently observed in elderly individuals or patients with concomitant cardiovascular diseases such as coronary artery disease and hypertension (1). The global prevalence of AF is approximately 0.5%, with an expected rate of up to 9% in individuals aged 65 years or older (2). The increasing incidence of AF poses a significant threat to public health and socioeconomic burden worldwide. Thromboembolism caused by AF is one of the most severe complications in patients with this condition.

The pathogenesis of AF is multifaceted, involving abnormalities in the conduction system, the effects of the autonomic nervous system, and abnormal ionic current metabolism in cardiomyocytes (3–6). Current research focuses on changes in ion channels, extracellular matrix, and intracellular signaling pathways. Risk factors for AF include Systemic arterial hypertension, coronary artery disease, heart failure, including heart failure with preserved ejection fraction, chronic kidney disease, obesity, obstructive sleep apnea (OSA) (7–9).

OSA is a prevalent sleep breathing disorder characterized by recurrent apnea and airway obstruction during sleep, resulting in decreased sleep quality and daytime sleepiness. The primary cause of OSA is the relaxation and collapse of the upper airway tissues, leading to airway narrowing or occlusion and subsequent apnea or shallow breathing (10). OSA is often associated with cardiovascular diseases such as obesity, hypertension, and diabetes (11). Recent studies have identified OSA as an independent risk factor for AF, with a close relationship between the two conditions (12). Patients with severe OSA have a two to five times higher risk of developing AF compared to normal individuals. Furthermore, sleep apnea has been linked to AF recurrence, particularly within the first year after recurrence (13). Untreated sleep apnea may exacerbate heart disease symptoms and increase the risk of heart disease, emphasizing the importance of sleep apnea interventions in patients with AF.

Obesity plays a crucial role in the pathogenesis of AF by adversely affecting cardiovascular hemodynamics and cardiac structure and function, thereby increasing the incidence of AF (14). It may also be associated with electroanatomical remodeling in obese patients. A meta-analysis on obesity and AF, which included 603,510 samples, demonstrated that obesity increases the likelihood of AF (15). Another study with medium and long-term follow-up results revealed that for every unit increase in body mass index (BMI), the risk of AF increased by 3% (95% CI, 1%–5%) (16). Obesity is also a significant risk factor for OSA, as it leads to the accumulation of fat in the neck, tongue, and larynx areas, causing airway obstruction.

In conclusion, AF is a prevalent cardiac arrhythmia with a multifaceted pathogenesis and various risk factors, including obstructive sleep apnea and obesity. The close relationship between these conditions highlights the importance of early intervention and management to reduce the incidence and recurrence of AF and its associated complications.

Mendelian randomization (MR) is an increasingly popular research method in the biomedical field. This method uses the random assignment pattern of human genes, or genetic variation, as an instrumental variable to reduce confounding factors, eliminate non-random errors, and improve the causal inference between exposure and outcome. As a result, MR can more accurately assess the effect of factors of concern on outcomes (17). MR has several advantages over traditional observational studies (18, 19). Therefore, this study used MR to investigate the relationship between OSA, obesity, and AF, with the hope of gaining new insights into interventions for the prevention of AF.

## 2 Methods and materials

### 2.1 Study design

To assess mediating effects, this study employed a two-step and multivariate MR approach. In the first step, we conducted MR studies using two-sample MR to examine the correlations between exposure and outcome, exposure and mediating variables, and mediating variables and outcome. If evidence of mediating effects was present in the two-step MR, we then employed multivariate Mendelian randomization (MVMR). Similar to traditional MR studies, the instrumentation of exposure and potential mediating variables is subject to three main assumptions. First, the instrument should be associated with the corresponding phenotype. Second, single nucleotide polymorphisms (SNPs) should not be confounded. Third, the association of SNPs with mediating variables and outcomes should occur only through their effects on exposure and mediating variables. Mediated MR results will be considered valid only if all three assumptions are met simultaneously.

### 2.2 Data sources

The genome-wide association study (GWAS) data for AF used in this study were obtained from a biobank-based study of European origin with more than one million participants. The study identified 111 independent risk variants at 142 loci associated with AF, using a total of 60,620 AF cases and 970,216 controls (20). The data were screened for genome-wide level significance (*p* < 5 × 10^−8^) and removal of linkage disequilibrium (*r*^2^ < 0.001).

The GWAS data for OSA and obesity were obtained from the FinnGen database, a comprehensive health data platform in Finland. The goal of FinnGen is to provide a large, high-quality data resource for medical research by collecting, integrating, and analyzing clinical, genomic, and health records of the Finnish population, the cut-off AF, OSA, obesity are classified according to the Tenth Revision of the International Classification of Diseases (ICD-10/ICD-9) published by the World Health Organization for the classification of diseases and health-related issues.

### 2.3 Statistical methods

In this study, we employed five different models, including inverse variance weighted (IVW), MR-Egger regression, weighted median, simple mode, and weighted mode, to explore causal correlations (21) and assess the causal relationships between exposure, mediators, and outcomes. The IVW method is the predominant MR analysis method used to combine the Wald ratios of individual SNPs (22). The MR-Egger method can be used to identify and correct for potential pleiotropy, and the weighted median method can provide consistent causal estimates when the highest 50% of the weights analyzed are from invalid instrumental variables. However, since the analysis was conducted assuming that all variants are valid instruments and instrumental variables may have directional pleiotropy (23), the estimates may be biased (24).

To assess whether the analysis is heterogeneous, we used Cochran's *Q*-values to assess the presence of heterogeneity. We also performed other sensitivity analyses, such as outlier test MR-PRESSO analysis, to identify and correct potential outliers and to avoid potential horizontal multicollinearity. Additionally, we used different conditioning plots, such as scatter plots, leave-one-out plots, and forest plots, to show the relationship between SNPs-exposure associations and SNPs-outcome associations, to reduce the effect of errors and improve the reliability and accuracy of the experiment and the contribution of each instrumental variable to the overall causal estimate.

## 3 Results

In this study, 8 SNPs were included in the MR study on obesity and AF. The results indicated a positive correlation between obesity and AF. In the IVW method, the association was significant [*p* = 1.38 × 10^−4^; OR, 95% Confidence Interval (CI) = 1.11 (1.05, 1.17)], in the MR Egger method, the association was not statistically significant [*p* = 1.72 × 10^−1^; OR, 95% CI = 1.15 (0.96, 1.36)], and in the Weighted median method, the results were significant [*p* = 1.41 × 10^−4^; OR, 95% CI = 1.11 (1.05, 1.17)].

For the study on obesity and obstructive sleep apnea (OSA), 8 SNPs were included. The results showed a positive correlation between obesity and AF. In the IVW method, the association was significant [*p* = 5.99 × 10^−11^; OR, 95% CI = 1.38 (1.25, 1.52)], in the MR Egger method, the association was significant [*p* = 3.40 × 10^−5^; OR, 95% CI = 1.57 (1.23, 1.76)], and in the Weighted median method, the results were significant [*p* = 7.80 × 10^−5^; OR, 95% CI = 1.47 (1.35, 1.60)].

**Figure 1:**
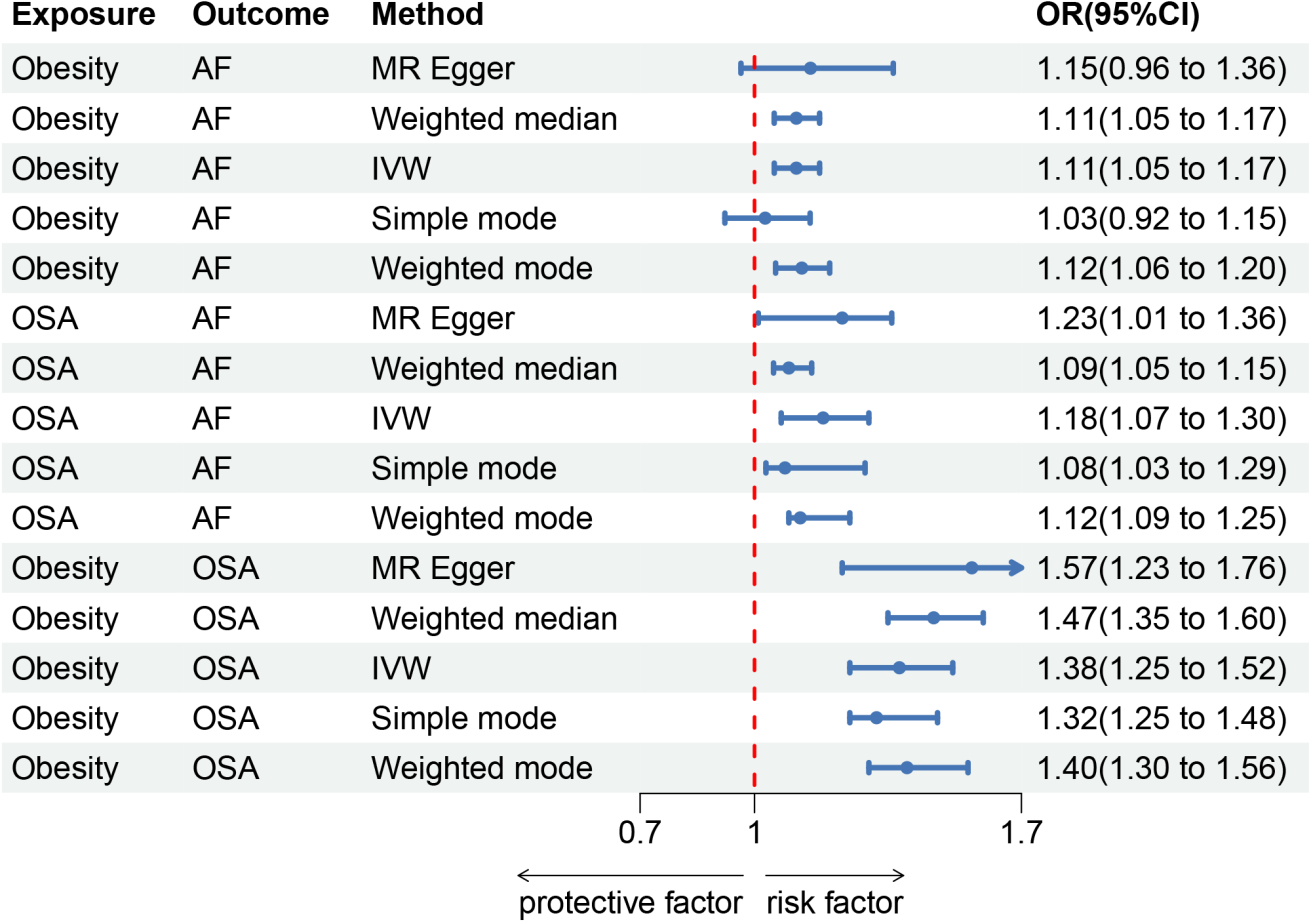
Forest map. (**A**) Obesity and AF forest map, (**B**) OSA and AF forest map, (**C**) Obesity and OSA forest map.

**Figure 2:**
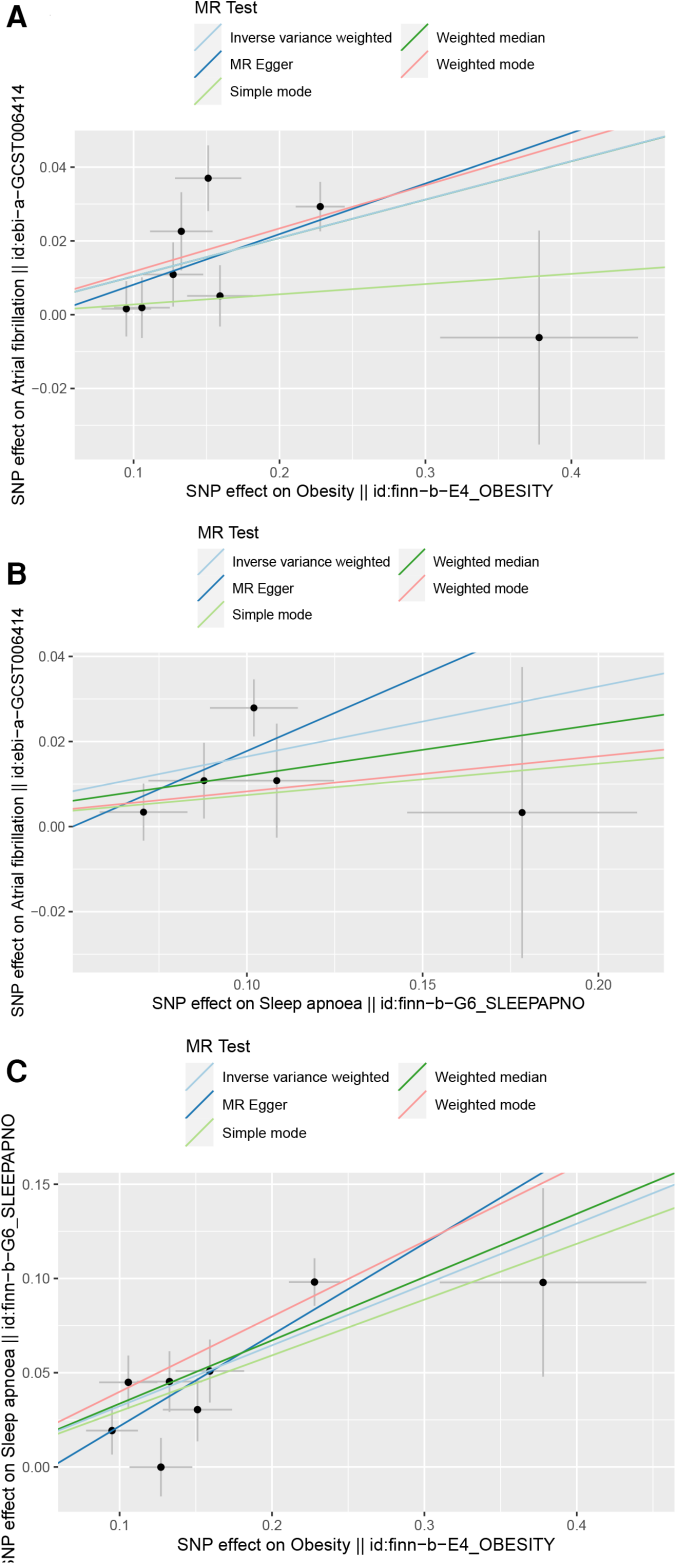
Scatter plot. (**A**) Scatter plot of obesity and AF, (**B**) Scatter plot of OSA and AF, (**C**) Scatter plot of obesity and OSA.

For the study on OSA and AF, 5 SNPs were included. The results indicated a positive correlation between OSA and AF. In the IVW method, the association was significant [*p* = 9.40 × 10^−5^; OR, 95% CI = 1.18 (1.07, 1.3)], in the MR Egger method, the association was significant [*p* = 6.50 × 10^−3^; OR, 95% CI = 1.23 (1.01, 1.36)], and in the Weighted median method, the results were significant [*p* = 5.60 × 10^−3^; OR, 95% CI = 1.05 (1.09, 1.15)]. The results are shown in Forest Plot, Figure 1. and the trend of MR results is shown in the scatter plot, Figure 2.

**Figure 3:**
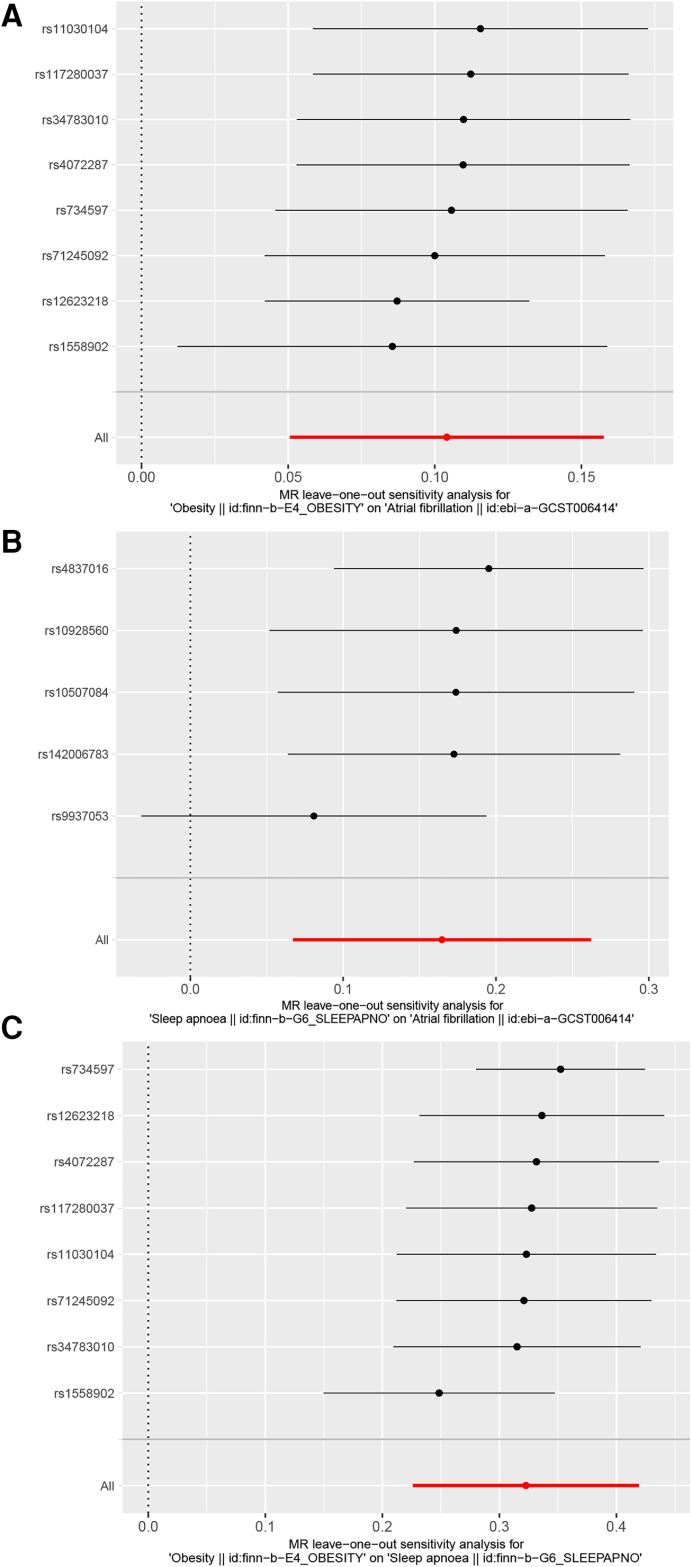
Leave-one-out plot. (**A**) Leave-one-out plot of obesity and AF, (**B**) Leave-one-out plot of OSA and AF, (**C**) Leave-one-out plot of obesity and OSA.

In the MR-Egger pleiotropy test, the intercept *p*-value was 0.71 for obesity and AF, indicating no pleiotropy bias in assessing the effect of obesity on AF using the IVW method. The intercept *p*-value was 0.271 for obesity and OSA, and in the sensitivity analysis of OSA and AF, the intercept *p*-value was 0.52 for the IVW method. Our results in the pleiotropy test were >0.05, and none of the data were confounded by pleiotropy. In the MR-PRESSO outlier test, *p* = 0.11 for obesity and AF, *p* = 0.13 for obesity and OSA, and *p* = 0.29 for OSA and AF, indicating no outliers were found in all three groups. There was no heterogeneity in Cochran's *Q*-test for obesity vs. AF (*p* = 0.051), obesity vs. OSA (*p* = 0.057), and OSA vs. AF (*p* = 0.26). Our results remained stable in the leave-one-out plot (Figure 3).

In the mediation effect analysis, we obtained an OSA mediation share of 6.4% by multivariate mediation MR. The results of the mediation analysis showed that OSA as a mediating variable has a role in the causal relationship of obesity on AF, and approximately 6.4% of the risk of obesity causing AF was due to OSA-mediated effects.

## 4 Discussion

We further conducted several sensitivity analyses to ensure the robustness of our findings. Outlier test MR-PRESSO analysis was performed to identify and correct potential outliers, and multiple validity tests were conducted to assess the reliability and accuracy of the experiment. We also used different conditioning plots, such as scatter plots, leave-one-out plots, and forest plots, to show the relationship between SNPs-exposure associations and SNPs-outcome associations, to reduce the effect of errors and improve the reliability and accuracy of the experiment.

In conclusion, our study provides evidence of a causal relationship between obesity and AF, with OSA serving as a mediating variable. Our findings suggest that reducing obesity and preventing or treating OSA may help prevent the development of AF. Further studies are needed to explore the mechanisms underlying this relationship and to develop effective interventions for the prevention and treatment of AF.

OSA can lead to increased cardiac load, which may contribute to the development of AF. The frequent apnea and hypopnea events in OSA patients can lead to repeated arousals from sleep, resulting in increased sympathetic activity, blood pressure, and heart rate (25). This can cause an increase in the left atrial pressure and volume, leading to atrial remodeling and increased susceptibility to AF (26). Furthermore, OSA can cause poor oxygenation and hemodynamic instability, which may contribute to the development of AF. The intermittent hypoxemia and hypercapnia in OSA patients can cause endothelial dysfunction, oxidative stress, and inflammation, which can lead to vascular damage and a prothrombotic state. This can increase the risk of thromboembolic events, which can lead to the development of AF (27).

Overall, the mechanism by which OSA causes AF is complex and multifactorial, involving alterations in cardiac electrophysiology and structure, autonomic nervous system disorders, increased cardiac load, poor oxygenation, and hemodynamic instability (28–30). Further research is needed to better understand the underlying mechanisms and develop effective interventions for the prevention and treatment of AF in OSA patients.

OSA may also contribute to the development of AF by increasing cardiac load, poor oxygenation, and hemodynamic instability. Patients with OSA are usually associated with cardiovascular diseases such as obesity, hypertension, and diabetes, which are themselves risk factors for AF (31). OSA in the treatment of AF may also reduce the efficacy of antiarrhythmic drugs, electrical cardioversion, and catheter ablation in the treatment of AF, thus increasing the recurrence rate of AF (32), and thus the outcome is poorer in patients with OSA combined with AF. This may be related to factors such as disturbances in the autonomic nervous system and alterations in cardiac electrophysiology and structure due to OSA (33).

In the relationship between obesity and AF, obesity has been shown to be an independent risk factor for AF in several studies (34), and the mechanism may involve several aspects such as alterations in cardiac structure and function, inflammatory response, and endocrine abnormalities (35). First, obesity can lead to structural and functional alterations in the heart, including atrial enlargement, myocardial fibrosis, and left ventricular hypertrophy. These alterations increase the risk of developing and recurring AF (36). A study of more than 25,000 patients with cardiovascular disease showed that the risk of AF increased by 20% for every 5-unit increase in BMI (37). Another study suggests that the effect of obesity on AF may be related to abdominal fat. This study found that abdominal fat area was positively associated with the incidence of AF. In addition, obesity can lead to inflammatory responses and endocrine abnormalities (38), factors that are also associated with the development and recurrence of AF. Obesity causes adipocytes to secrete a variety of inflammatory factors, including tumor necrosis factor-α (TNF-α), interleukin-6 (IL-6), and C-reactive protein (CRP) (39). These factors can lead to myocardial fibrosis and electrophysiological abnormalities, which can increase the risk of AF (40). Obesity can also lead to endocrine abnormalities, such as insulin resistance and abnormal insulin secretion, and these factors are also associated with the development and recurrence of AF (41, 42). Finally, obesity also affects the outcome and prognosis of treatment for AF. One study found that obesity decreased the response of patients with AF to antiarrhythmic drug therapy, thereby increasing the risk of recurrence (43, 44).

Moreover, the MR approach used in this study assumes that the genetic variants used as instrumental variables are only related to the exposure variable and not to any confounding factors or other pathways that may affect the outcome variable. However, there may still be pleiotropic effects of these genetic variants, which can lead to biased estimates of causal effects. Therefore, further validation and replication of the findings are needed using other methods and data sources.

In conclusion, our study underscores the potential impact of obesity and OSA on the occurrence of AF, which holds significant implications for clinical practice. For example, clinical interventions such as weight reduction and the prevention or treatment of OSA may aid in mitigating the risk of AF occurrence and recurrence in patients. These considerations can be integrated into patient management plans, particularly for individuals already diagnosed with AF or deemed at risk for the condition. Early screening, especially for obese and OSA patients, including those with cardiovascular disease risk factors, can be beneficial as it may help prevent the onset of AF through timely interventions. Moreover, personalized treatment is paramount: comprehending the interplay between obesity, OSA, and AF can empower clinicians to tailor treatment plans that optimize cardiovascular health management for patients. Lastly, further research is imperative: this study sets the stage for deeper exploration of the relationships between obesity, OSA, and AF. Subsequent investigations can delve into the biological mechanisms underpinning these associations and devise more efficacious intervention strategies.

## 5 Conclusion

This study provides new insights into the potential role of OSA as a mediating variable in the relationship between obesity and AF, and highlights the importance of addressing obesity as a modifiable risk factor for both OSA and AF. Interventions aimed at reducing obesity may have a beneficial effect on reducing the incidence of OSA and AF. However, further studies are needed to explore the optimal interventions for the prevention and treatment of obesity, OSA, and AF, and to identify potential targets for therapeutic interventions.

## Data Availability

The datasets presented in this study can be found in online repositories. The names of the repository/repositories and accession number(s) can be found in the article/Supplementary Material.
